# Supplementary material for: Serum Vitamin D Concentrations, Time to Pregnancy, and Pregnancy Outcomes among Preconception Couples: A Cohort Study in Shanghai, China
Source: Nutrients. 2022 Jul 26;14(15):3058. doi: 10.3390/nu14153058 (PMC9330297; doi:10.3390/nu14153058)
Supplement: Supplementary file 1 [file nutrients-14-03058-s001.zip › nutrients-1824013-supplementary.pdf]

**Supplementary Table S1: Associations of preconception and pregnancy vitamin D levels with clinical pregnancy, time to pregnancy and pregnancy outcomes**

| Outcomes | Sufficient 25(OH)D levels (≥30 ng/ml) vs insufficient 25(OH)D levels (<30 ng/ml) | | | |
|---|---|---|---|---|
| | 25(OH)D levels among preconception women | 25(OH)D levels among preconception men | 25(OH)D levels during the 2nd trimester of the pregnancy | 25(OH)D levels during the 3rd trimester of the pregnancy |
| **Clinical pregnancy within six months** | | | | |
| cOR(95%CI) | 0.92(0.36~2.31) | 3.15(1.06~9.39)* | -- | -- |
| aOR(95%CI)[a] | 0.90(0.32~2.54) | 3.72(1.16~11.86)* | -- | -- |
| **Time to pregnancy** | | | | |
| cFR(95%CI) | 1.08(0.71~1.62) | 1.32(0.92~1.90) | -- | -- |
| aFR(95%CI)[a] | 1.08(0.70~1.69) | 1.50(1.01~2.23) | -- | -- |
| **Gestational diabetes mellitus** | | | | |
| cOR(95%CI) | 1.38(0.43~4.42) | -- | 0.85(0.24~3.07) | 2.57(0.91~7.22) |
| aOR(95%CI)[b] | 2.23(0.60~8.37) | -- | 0.87(0.21~3.55) | 2.26(0.72~8.88) |
| **Gestational anemia** | | | | |
| cOR(95%CI) | 0.99(0.41~2.44) | -- | 1.08(0.46~2.53) | 0.26(0.07~0.89)* |
| aOR(95%CI)[b] | 1.23(0.47~3.21) | -- | 1.28(0.51~3.23) | 0.22(0.06~0.82)* |
| **Premature rupture of membranes** | | | | |
| cOR(95%CI) | 0.67(0.15~3.07) | -- | 0.28(0.04~2.16) | 0.30(0.04~2.37) |
| aOR(95%CI)[b] | 0.89(0.17~4.67) | -- | 0.25(0.03~2.10) | 0.22(0.02~1.99) |
| **Delivery gestational age** | | | | |
| $\beta$(95%CI)[b] | -0.06(-0.54~0.41) | -- | -0.07(-0.54~0.40) | 0.53(0.05~1.01)* |
| **Ponderal index** | | | | |
| $\beta$(95%CI)[b] | 0.01(-0.08~0.10) | -- | -0.01(-0.10~0.08) | 0.10(0.01~0.19)* |

Abbreviations: cOR, crude odds ratio; aOR, adjusted odds ratio; CI, confidence interval; cFR, crude fecundability ratio; aFR, adjusted fecundability ratio.

[a]Adjusted for preconception age, preconception BMI, education, smoking, alcohol consumption, gravidity, multivitamin supplementation, calcium supplementation, folic acid supplementation, taking frequent milk intake before pregnancy, and season of blood sample collection.

[b]Adjusted for women's age and BMI, education, household annual income per capita, gravidity, weight gain during pregnancy, multivitamin supplementation, calcium supplementation and folic acid, and taking frequent milk and deep-sea fish foods during pregnancy.

**P*<0.05.
